# Neutrophils cultured ex vivo from CD34^+^ stem cells are immature and genetically tractable

**DOI:** 10.1186/s12967-024-05337-x

**Published:** 2024-05-31

**Authors:** Claire A. Naveh, Kiran Roberts, Przemysław Zakrzewski, Christopher M. Rice, Fernando M. Ponce-Garcia, Kathryn Fleming, Megan Thompson, Nawamin Panyapiean, Huan Jiang, Stephanie Diezmann, Pedro L. Moura, Ashley M. Toye, Borko Amulic

**Affiliations:** 1 School of Cellular and Molecular Medicine, Biomedical Sciences Building, University of Bristol, Bristol, BS8 1TD UK; 2 School of Biochemistry, Biomedical Sciences Building, University of Bristol, Bristol, BS8 1TD UK; 3 Center for Hematology and Regenerative Medicine, Department of Medicine Huddinge (MedH), Karolinska Institutet, Huddinge, Sweden

**Keywords:** Neutrophil, Granulopoiesis, Ex vivo differentiation, CD34^+^stem cells, Proteomics

## Abstract

**Background:**

Neutrophils are granulocytes with essential antimicrobial effector functions and short lifespans. During infection or sterile inflammation, emergency granulopoiesis leads to release of immature neutrophils from the bone marrow, serving to boost circulating neutrophil counts. Steady state and emergency granulopoiesis are incompletely understood, partly due to a lack of genetically amenable models of neutrophil development.

**Methods:**

We optimised a method for ex vivo production of human neutrophils from CD34^+^ haematopoietic progenitors. Using flow cytometry, we phenotypically compared cultured neutrophils with native neutrophils from donors experiencing emergency granulopoiesis, and steady state neutrophils from non-challenged donors. We carry out functional and proteomic characterisation of cultured neutrophils and establish genome editing of progenitors.

**Results:**

We obtain high yields of ex vivo cultured neutrophils, which phenotypically resemble immature neutrophils released into the circulation during emergency granulopoiesis. Cultured neutrophils have similar rates of ROS production and bacterial killing but altered degranulation, cytokine release and antifungal activity compared to mature neutrophils isolated from peripheral blood. These differences are likely due to incomplete synthesis of granule proteins, as demonstrated by proteomic analysis.

**Conclusion:**

Ex vivo cultured neutrophils are genetically tractable via genome editing of precursors and provide a powerful model system for investigating the properties and behaviour of immature neutrophils.

**Supplementary Information:**

The online version contains supplementary material available at 10.1186/s12967-024-05337-x.

## Background

Neutrophils are the most abundant and potent antimicrobial effector cells in humans. They combat bacterial and fungal pathogens using an arsenal of antimicrobial responses, including phagocytosis, reactive oxygen species (ROS) production, degranulation and release of extracellular chromatin decorated with antimicrobial peptides, termed neutrophil extracellular traps (NETs) [1]. Patients with congenital neutropenia, who have abnormally low circulating neutrophils counts, succumb to severe bacterial and fungal infections [2, 3]. In contrast, excessive or dysregulated neutrophil activity promotes pathology in sepsis [4] and malaria [5–7], as well as in non-infectious diseases such as cancer [8], autoimmunity [9] and cardiovascular disease [10]. Regulation of neutrophil development and function is therefore essential for health.

Neutrophils develop in the bone marrow from CD34-expressing (CD34^+^) granulocyte-monocyte progenitors (GMPs) [11]. This process, termed granulopoiesis, produces an estimated 10^11^ neutrophils daily in healthy individuals [12]. Development from the committed proliferative myeloblast to release of mature neutrophils into circulation takes approximately 14 days [13], during which progenitors gradually acquire cytoplasmic granules, lobulated nuclei and ROS producing enzymes such as NADPH oxidase (NOX2) and myeloperoxidase (MPO). Granulopoiesis is driven by the growth factors granulocyte colony stimulating factor (GCSF) [14, 15] and granulocyte–macrophage colony-stimulating factor (GM-CSF) [16, 17]. In addition to promoting differentiation in the bone marrow, GCSF promotes mobilisation of mature and immature neutrophils out of the bone marrow and into the circulation, where it also extends their lifespan [18, 19].

Neutrophils were traditionally considered to be homogenous cells. This view has recently been challenged, with reports of various activation and differentiation states, both in healthy individuals [20] and during inflammation [8, 12]. One of the main determinants of neutrophil phenotype is maturity. During inflammation, elevated GCSF production promotes the release of immature neutrophils from the bone marrow. This phenomenon has been observed for decades in clinical settings, where the presence of immature morphological features in circulating neutrophils is termed ‘left shift’. Flow cytometry studies of patient blood can identify immature neutrophils by reduced expression of surface maturity markers such as CD10 and CD101, relative to mature neutrophils [21]. Strikingly, the number of circulating immature neutrophils is often associated with poor prognosis in a variety of autoimmune and inflammatory diseases such as systemic lupus erythematosus (SLE), COVID-19 and late-stage cancer [21–27]. Despite this strong association with severe disease, the function and inflammatory potential of immature neutrophils remains unclear.

GCSF administration is sufficient to mobilise immature neutrophils into the circulation; this was demonstrated in GCSF-treated allogeneic stem cell donors (GCSF-D) [28]. Importantly, these studies suggested functional differences between control and GCSF-D neutrophils, including elevated production of proinflammatory cytokines, reduced motility and capacity to produce ROS, as well as reduced ability to suppress the fungal pathogen *Candida albicans (C. albicans*) [19, 28–30].

Existing models for investigating neutrophils are limited. Peripheral blood neutrophils are very short lived [31], precluding genetic manipulation, while myeloid cell lines such as HL-60 have important functional deficiencies, including absence of secondary granules [32]. To facilitate studies of neutrophil development, we optimised an ex vivo protocol for differentiation of neutrophils from human hematopoietic stem and progenitor cells (HSPCs). Previous culture protocols have described neutrophil differentiation from diverse sources such as embryonic stem cells, induced pluripotent stem cells as well as bone marrow and peripheral blood HSPCs [33–45]; these studies report that cultured neutrophils resemble native neutrophils in nuclear morphology, surface marker expression and some neutrophil effector functions such as ROS production, bacterial killing, phagocytosis and chemotaxis. Based on these reports, we optimised the culture conditions to obtain a high yield of CD34^+^ HSPC-derived neutrophils. We show that cultured neutrophils more closely resemble immature neutrophils than steady state circulating neutrophils, and that they are amenable to genome editing, enabling mechanistic studies of immature neutrophil function.

## Results

### Optimisation of a neutrophil culture and differentiation protocol from haematopoietic stem cells

**Fig. 1:**
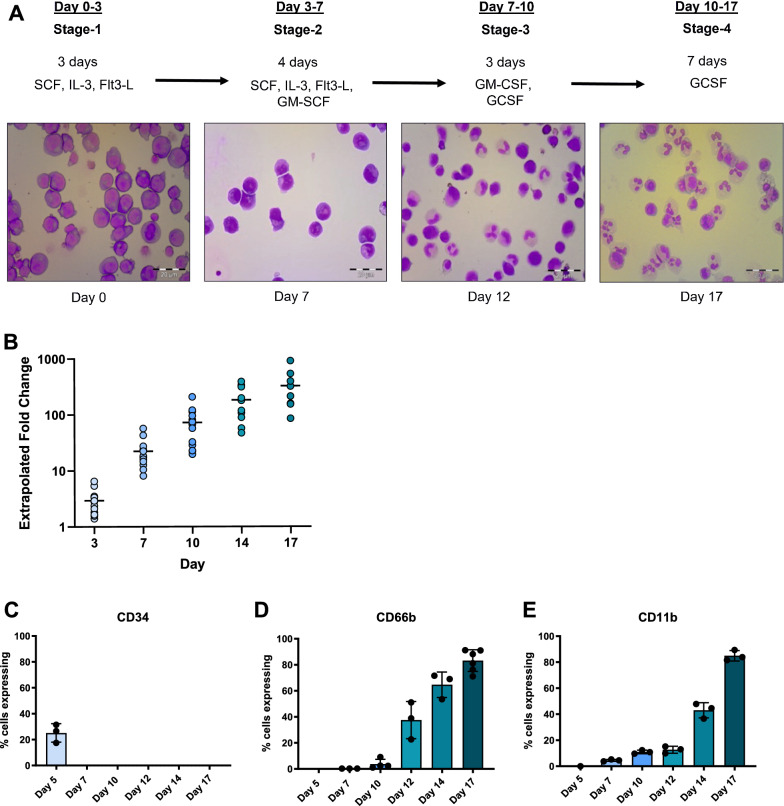
Optimisation of neutrophil culture protocol . **A** Representative Wright Giemsa-stained cytospin images of neutrophil differentiation steps, with cytokine protocol. Size bar = 20 micron. **B** Extrapolated fold expansion of cultured total cell count over 17 days of differentiation, n = 7–11. **C**–**E** Surface marker expression of HSPC marker CD34 (**C**) and granulocyte markers CD66b (**D**) and CD11b (**E**) over days 5–17 of differentiation, n = 3–6

We isolated CD34^+^ HSPCs from apheresis cones using immunomagnetic selection (Fig. S1A) and cultured these with multiple combinations of stem cell proliferation and expansion factors (stem cell factor (SCF), interleukin-3 (IL-3) and fms-like tyrosine kinase 3 ligand (Flt3-L)), followed by neutrophil differentiation factors (GM-CSF and G-CSF). We tested a range of reported differentiation conditions [33, 35, 37, 40, 43, 46–48] to generate our optimised differentiation protocol shown in Fig. 1A, which was selected based on superior yield, differentiation efficiency and viability of cells. This protocol resulted in an average 326 ± 248 fold expansion (n = 7–11, Fig. 1B) with a loss of CD34 expression after day 5 (Fig. 1C) and progressive increase after day 7 of granulocyte marker expression (CD66b and CD11b, Fig. 1D and E respectively). Neutrophil nuclear lobulation and surface markers peaked at differentiation day 17 (Fig. 1A, C-E and Fig. S1C), yielding an average of 75.45% neutrophils co-expressing the markers CD66b and CD15 (n = 4, Fig. S1B), with over 80% viability at the end of the differentiation protocol (Fig. S1F).

### Cultured neutrophils phenotypically resemble GCSF-mobilised immature neutrophils

**Fig. 2:**
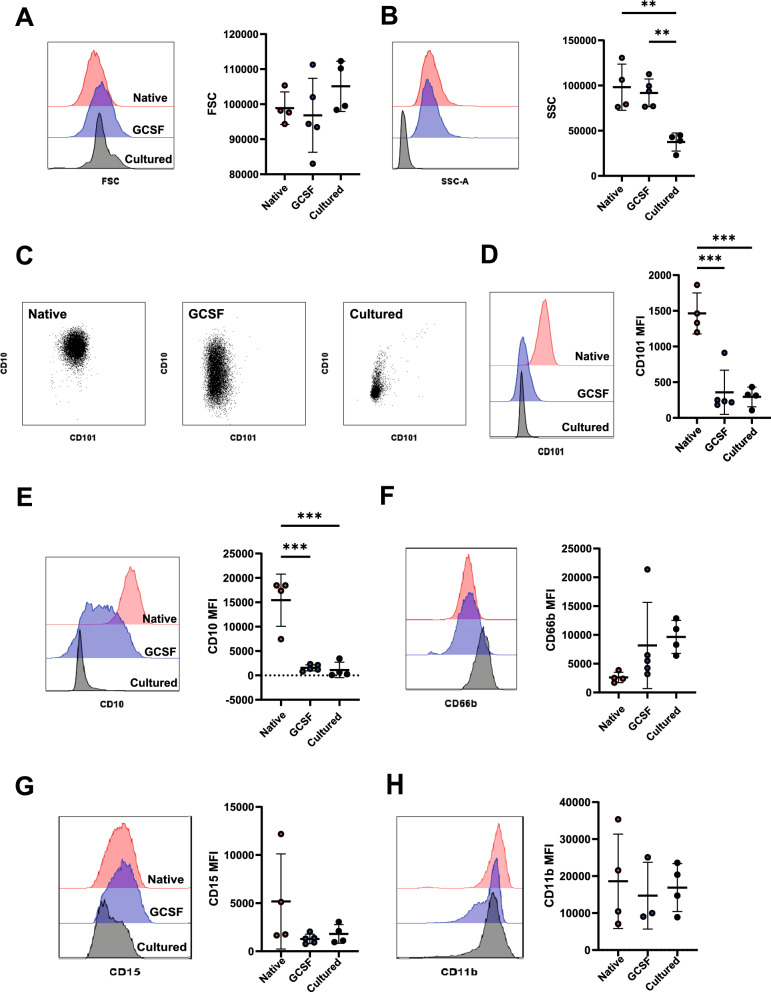
Flow cytometry analysis of surface markers on native, GCSF-D and cultured neutrophils. **A**, **B** Representative histogram and quantification of forward scatter (FSC) (**A**) and side scatter (SSC) (**B**). **C** Representative scatter dot plots displaying CD101 and CD10 expression on native, GCSF-D and cultured neutrophils. **D**–**H** Representative histograms and mean fluorescent intensity (MFI) quantifications of CD101 (**D**), CD10 (**E**), CD66b (**F**), CD15 (**G**) and CD11b (**H**). Histograms colour coded as: native (red), GCSF-D (blue) and cultured (black) neutrophils. Data were analysed by one-way ANOVA with Tukey’s multiple comparisons displayed on graph, n = 3–5, *p < 0.05, **p < 0.01, ***p < 0.001

GCSF treated healthy donors (GCSF-D) are known to have a significant population of immature neutrophils circulating in peripheral blood [49]. We used flow cytometry to compare profiles of cultured, GCSF-D and steady state native neutrophils isolated from peripheral blood (gating strategy shown in Fig. S2A). GCSF-D and steady state native neutrophils had similar forward (FSC) and side scatter (SSC) (Fig. 2A–B), indicating similar size and granularity. Cultured neutrophils displayed similar FSC but reduced SSC indicating decreased granularity, possibly due to reduced abundance of cytoplasmic vesicles. As expected, GCSF-D neutrophils had significantly lower expression of maturity markers CD10 and CD101 compared to native neutrophils (Fig. 2C–E, Fig. S2B–D). Cultured neutrophils also had a reduced abundance of maturity markers, indicating immature status. In contrast, we found similar levels of granulocyte markers in all three cell types (Fig. 2F–H, Fig. S2E–G), with a trend for increased CD66b in cultured neutrophils. In summary, based on their surface marker expression, neutrophils cultured from CD34^+^ HSPCs phenocopy immature GSCF-D neutrophils.

### Functional comparison of cultured and native neutrophils

**Fig. 3:**
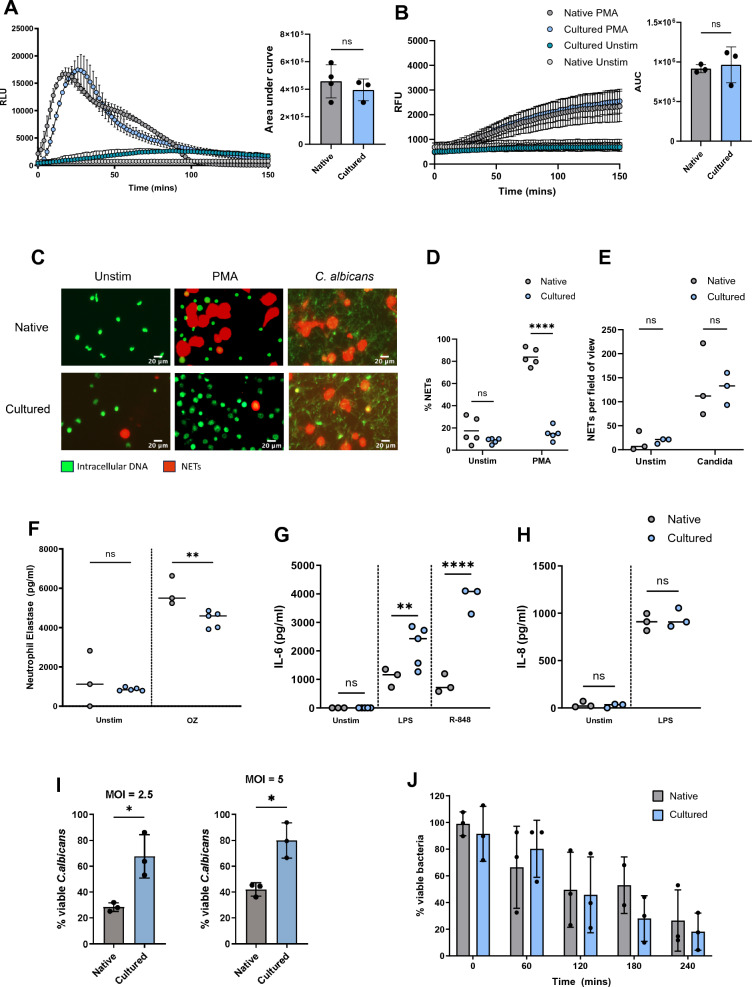
Comparison of effector functions in cultured and native neutrophils. **A**, **B** Detection of ROS with luminol (**A**) and APF (**B**) in native or cultured neutrophils stimulated with 100 nM PMA. Left: A representative kinetic plot of the respiratory burst in individual donors; right: area under the curve (AUC) quantification, n = 3. **C** Representative images of NETs induced with 100 nM PMA and *C. albicans* (MOI = 5, 4 h), stained with SYTO green and SYTOX orange. **D** Quantification of average percent NETs per field of 10X view in response to PMA induction (n = 2–4). **E** Quantification of average NET frequency per 10X field of view, induced by *C. albicans* n = 3 donors. **F** Exocytosis of NE in response to stimulation with 25 µg/ml opsonised zymosan (OZ) for 1 h, quantified by ELISA, n = 3–5. **G**-**H** IL-6 (**G**) and IL-8 (**H**) cytokine release from native and cultured neutrophils stimulated overnight with 100 ng/mL LPS or 5 µM R-848, quantified by ELISA, n = 3–5. **I**: Viability of opsonised *C. albican*s after incubation with native or cultured neutrophils for 2.5 h at MOI 2.5 (left) or 5 (right), relative to non-treated *C. albicans*, n = 3. J Viability of opsonised *S. aureus* JE2 after incubation with native or cultured neutrophils at MOI = 5, over 240 min (all non-significant, n = 3). Error bars indicate mean ± standard deviation. Data were analysed by two tailed Students t test, *p < 0.05, **p < 0.01, ***p < 0.001

Immature peripheral blood neutrophils are reported to have reduced oxidative burst, impaired capacity to kill *C. albicans* and increased cytokine production to toll-like receptor (TLR) agonists [28–30]. We compared effector responses in cultured neutrophils and steady state native neutrophils isolated from peripheral blood, to explore any possible functional differences. We observed no difference in total ROS production, measured by luminol, in response to phorbol myristate acetate (PMA), a protein kinase C (PKC) agonist, when analysed by area under the curve (AUC) (Fig. 3A). The luminol experiment did however reveal subtle kinetic differences in the ROS response (Fig. 3A), potentially indicating differences in in antioxidant response or NOX2 assembly. ROS detection with aminophenyl fluorescein (APF), a dye that detects peroxynitrites and MPO-catalysed hypochlorous acid, also showed comparable production of intracellular ROS (Fig. 3B). In summary, NOX2 and MPO activity are similar in cultured and native neutrophils.

Next, we measured the ability of cultured neutrophils to release NETs in response to the fungal pathogen *C. albicans* and phorbol-12-myristate-13-acetate (PMA). NETs were stained with SYTOX Orange dye, which labels extracellular DNA, while intact neutrophils were detected with SYTO Green. Cultured neutrophils did not engage in NET formation in response to PMA at the analysed timepoint (4 h) (Fig. 3C and D); however the response to *C. albicans* was equivalent to native neutrophils (Fig. 3C and E). These two stimuli are known to engage different NETosis pathways: while PMA is entirely NOX2-dependent, *C. albicans* is partially independent of the oxidase [50]. We therefore tested another NOX2-independent stimulus: the calcium ionophore A23187, which induced comparable levels of NETs in both native and cultured neutrophils (Fig. S2H and I). In conclusion, cultured neutrophils can engage in NOX2-independent NETosis but are impaired in the NOX2-dependent pathway induced by PMA.

Next, we measured exocytosis of primary granules by quantifying neutrophil elastase (NE) release in response to stimulation with serum-opsonized zymosan (OZ), a fungal cell wall component. We observed a 27% reduction in extracellular NE release in stimulated cultured neutrophils compared to native ones (Fig. 3F), suggesting either reduced NE expression or decreased propensity to degranulate in response to OZ.

As reported for immature neutrophils from GCSF-D, production of proinflammatory cytokine interleukin-6 (IL-6) was significantly elevated in cultured neutrophils in response to both TLR4 agonist lipopolysaccharide (LPS) and TLR7/8 agonist resiquimod (R-848) (Fig. 3G). However, no difference in IL-8 production was detected in response to LPS (Fig. 3H).

Lastly, we measured the ability of cultured neutrophils to suppress proliferation of two important human pathogens. We observed that cultured neutrophils were able to kill *C. albicans,* however this ability was reduced by 50% compared to native neutrophils (normalised to nontreated *C. albicans* control; Fig. 3I). In contrast, cultured neutrophils were able to suppress growth of a clinical isolate of the Gram-positive bacterium *Staphylococcus aureus* (*S. aureus*) at rates comparable to those observed with native neutrophils (Fig. 3J).

### Cultured and native neutrophils have distinct proteomes

**Fig. 4:**
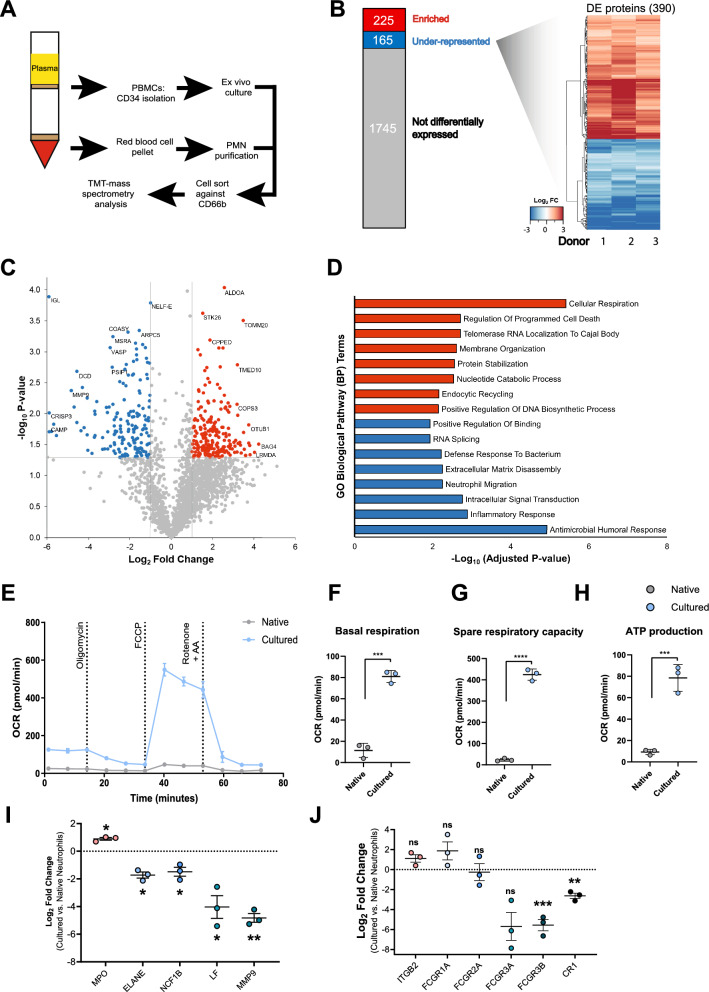
Mass spectrometry comparison of cultured and native neutrophil proteomes. **A** Experimental design for TMT proteomic analysis. **B** Stacked bar chart and heat map displaying significantly enriched proteins (red), significantly under-represented (blue) and unaltered proteins (grey), using native neutrophils as the baseline for comparison. Differentially expressed (DE) proteins were displayed using heatmap visualisation of Log2 Fold Change (Log2FC) values of both significantly enriched and underrepresented proteins. **C** Volcano plot displaying total and differentially expressed proteins (P-value threshold of 0.05, absolute Log2FC threshold of 1.00). **D** Top 8 unique Gene Ontology (GO) terms resulting from gene list enrichment analysis of enriched (red) and under-represented (blue) proteins using the GO Biological Pathway module. **E** Representative Seahorse metabolic flux analyser mitochondrial stress test of native and cultured neutrophils treated with oligomycin, FCCP and rotenone/antimycin A. **F**–**H** Oxygen consumption rate (OCR) of native and cultured neutrophils measuring basal respiration (**F**), spare respiratory capacity (**G**) and ATP production (**H**). Error bars indicate mean ± standard deviation (n = 3 differentiations), two tailed Students T test (***P < 0.001, ****P < 0.001). **I**, **J** Normalised abundances of key granule proteins (**I**) and opsonic surface receptors (**J**), n = 3 differentiations, (*p < 0.05, **p < 0.001, ***p < 0.0001)

To investigate what underpins the functional differences described above, we analysed the proteomes of cultured and steady state native neutrophils, using tandem mass tag (TMT) mass spectrometry. Cultured and native neutrophils were obtained from the same donors, allowing for matched proteomic analysis. Prior to mass spectrometry, both native and cultured neutrophils were FACS sorted for CD66b^+^ to eliminate contamination with precursors or other cell types (Fig. 4A, Fig. S3A). We applied a stringent false discovery rate (FDR) filtering step of ≤ 1%, which led to identification of 2359 proteins. A large majority, 74% of detected proteins (1745 in total), were unchanged between cultured and native neutrophils (Fig. 4B, Supplementary File 1). Enriched (red) and underrepresented (blue) proteins were defined by an absolute log2 fold change (Log2FC) of at least 1 and comparison p-value < 0.05. Enriched and underrepresented proteins were altogether consistent among all donors as visualised by heatmap (Fig. 4B) and volcano plot (Fig. 4C) of all differentially expressed proteins. Despite the change in relative abundances of proteins, only 12 proteins were exclusively detected in cultured neutrophils and not in native cells (Supplementary Table 1), although it remains unclear if these are functionally relevant.

To identify differentially regulated pathways, we conducted gene ontology (GO) term analysis on enriched and underrepresented protein sets (Fig. 4D) with the entire human proteome set as the background. This identified ‘cellular respiration’ as the top enriched pathway in cultured cells, indicating an altered metabolic state. Similarly, both ‘protein stabilisation’ and ‘nucleotide catabolic process’ were among the top 6 enriched pathways, further suggesting that biosynthetic pathways are altered in cultured neutrophils, a finding consistent with the fact that enhanced mitochondrial respiration and biosynthesis are both more prominent in neutrophil precursors, compared to mature neutrophils [51]. On the other hand, multiple antimicrobial pathways were downregulated in cultured neutrophils, including ‘antimicrobial humoral response’, ‘inflammatory response’ and ‘defence response to bacterium’.

To further interrogate specific molecular pathways altered between cultured and native neutrophils, we also carried out Reactome pathway analysis. As with the GO analysis, the top enriched Reactome pathway in native neutrophils was ‘TCA Cycle and respiratory electron transport’ (Fig. S3B), again identifying an enrichment of mitochondrial proteins in immature cultured neutrophils. Moreover, we found that individual proteins important for the TCA cycle and respiratory electron transport were enriched (Fig. S3C). We confirmed enhanced mitochondrial activity in cultured neutrophils using the Seahorse metabolic flux analyser mitochondrial stress test, which showed increased basal oxygen consumption rate (OCR), spare respiratory capacity and mitochondrial ATP production (Fig. 4E–H). STRING analysis of the 225 overexpressed proteins also highlighted a cluster of diverse mitochondrial proteins (highlighted in blue, Fig. S3E). Mitochondrial respiration is a hallmark of immature neutrophils circulating in inflammatory disease such as COVID-19 and cancer [21, 52].

Several granule proteins stood out as significantly reduced in cultured neutrophils, including matrix metalloprotease 9 (MMP9), cysteine rich secretory protein 3 (CRISP3), cathelicidin antimicrobial peptide (CAMP) (Fig. 4C, I, Supplementary File 1), supporting our findings on reduced SSC and reduced degranulation in cultured versus native cells. Reactome analysis also identified the ‘innate immune response’ and ‘neutrophil degranulation’ as the most underrepresented pathways in cultured neutrophils (Fig. S3B). Neutrophil degranulation was enriched in both overrepresented and underrepresented proteins, albeit more strongly in the underrepresented protein set (Fig. S3E and S3F), so we further investigated granule protein abundance. Indeed, comparison of the individual abundance of representative granule proteins highlighted significant reduction of core granule protein abundance, with the exception of MPO (Fig. 4I). These changes mirrored trends observed in a published proteomic comparison of GCSF-D and steady state native neutrophils (Figure S3D), which also detected reductions in key secondary granule proteins in mobilised neutrophils [53]. Collectively, these data argue for a significant perturbation of granule protein synthesis in cultured neutrophils, which is supportive of our suggestion that they have not reached complete maturity. Cultured neutrophils also demonstrated reduced expression of FcyRIIIB (CD16B), the main receptor for antibody opsonised *C. albicans*, potentially explaining the impaired fungal killing (Fig. 4J).

### CRISPR/Cas9 genome editing of cultured neutrophils

**Fig. 5:**
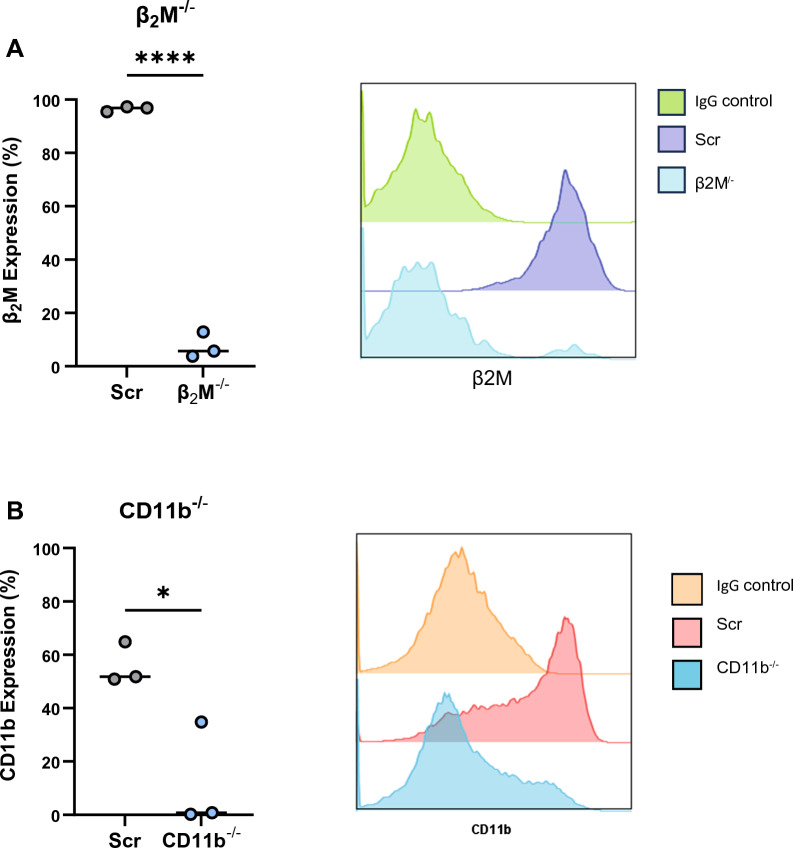
Genome editing of cultured neutrophils. **A**, **B** CRISPR/Cas9 mediated knockout of β_2_M (**A**) and CD11b (**B**) demonstrated by flow cytometric quantification of the percentage of cells expressing targeted protein (left) and representative histograms of differentiated neutrophils (right) at day 17 of differentiation, n = 3. Data were analysed by two tailed Students t test, *p < 0.05, **p < 0.01, ***p < 0.001, ****p < 0.0001

Neutrophils are notoriously short lived and difficult to transfect, therefore the ability to culture them from CD34^+^ stem cells offers a window of opportunity to explore gene editing [54]. We investigated whether cultured neutrophil precursors are amenable to genetic manipulation, prior to differentiation, as a tool for modifying gene expression in immature neutrophils. We targeted CD34^+^ HSPCs (day 3 of culture) and used nucleofection to deliver ribonucleoproteins (RNPs) of Cas9 and guide RNA (gRNA). We used two different gRNAs, targeting β_2_ microglobulin (β_2_M), a transmembrane protein expressed on all nucleated cells, and CD11b, an integrin expressed on myeloid cells. Nucleofection was efficient and did not require selection, with differentiated neutrophils demonstrating 92.5% and 88.1% loss of β2M (Fig. 5A) and CD11b (Fig. 5B) surface expression, respectively, at culture endpoint (day 17). We found no significant difference in viability of the gene-edited cells (Fig. S4A). Moreover, CD66b expression and therefore neutrophil differentiation was not affected (Fig. S4B), confirming that modification of precursors by CRISPR/Cas9 is well tolerated and can be used as a molecular tool to investigate immature neutrophils.

## Discussion

Many basic questions in neutrophil biology remain unanswered, despite the importance of these cells in antimicrobial defence and inflammatory disease. This is in part because neutrophil research is hampered by a lack of tools for genetic manipulation and by the short lifespan of neutrophils isolated from peripheral blood. One important outstanding question is the existence of neutrophil heterogeneity in disease conditions and whether neutrophils produced during inflammation differ from those produced at steady state. Studies using single cell RNA sequencing are providing compelling evidence for the presence of multiple neutrophil states [20]. Unsurprisingly for a cell with a short circulating lifespan (1–5 days in circulation) [1], neutrophil maturity is emerging as a major factor in determining phenotype and function.

Building on previous published work in the field, we developed an improved method for ex vivo culture and genetic manipulation of neutrophils. Our optimised protocol uses CD34^+^ stem cells isolated from waste apheresis cones, rather than the more restricted and costly embryonic or induced pluripotent stem cells, or HSPCs isolated from cord blood or bone marrow [34, 35, 40–43]. Compared to previous reports [33, 35, 37, 40, 43, 46–48], our protocol offers the combined advantages of (1) a shortened 17-day differentiation, (2) high levels of purity and (3) high yield. Most recently, Kuhikar et al. described neutrophil differentiation from apheresis cones, showing an average 72.4-fold expansion, with 57.37% of neutrophils expressing CD66b and 70.48% expressing CD15 [55]. Our protocol achieves an average 326-fold expansion with 75.45% of neutrophils co-expressing CD66b and CD15. The yields obtained using this culture method are robust, however we observed there was significant variation in yield between the apheresis blood donors. We currently do not know the reason for this but assume it is natural biological variation in the ability of donor CD34^+^ stem cells to proliferate. Interestingly, Jie et al. were able to achieve significantly higher expansion rates (~ 490,000), using cord blood CD34^+^cells [34], confirming that cord blood HSPCs have higher proliferative potential [48]. While this study did not carry out extensive characterization of cultured neutrophils, it did demonstrate intact bactericidal activity, motility as well as viability after transfusion into immunodeficient mice [34].

Quantification of expression of the well-established maturity markers CD10 and CD101 demonstrated that cultured neutrophils phenotypically resemble immature neutrophils mobilised by GCSF administration. Similarly to GCSF-D neutrophils [19], cultured neutrophils demonstrated reduced *C. albicans* killing and overproduction of IL-6, compared to steady state native cells. The overproduction of IL-6 may be a potential pathogenic mechanism in diseases where immature neutrophils are implicated. Indeed, secretion of IL-6 by immature neutrophils has been implicated in autoinflammatory diseases such as chronic graft versus host disease and adult-onset Still’s disease [56, 57]. Future studies will investigate signaling pathways active in GCSF-D and cultured neutrophils, in order to understand how GCSF alters their functional properties, compared to mature peripheral blood neutrophils.

Cultured neutrophils produce an efficient NOX2 oxidative burst, as previously shown [35, 36, 39, 43, 48, 58], and have comparable levels of MPO activity to native cells. Interestingly MPO was enriched in the proteome of cultured cells, reflecting reports of elevated MPO expression in immature CD10^−^ neutrophils circulating in myocardial infarction patients [59].

Our proteomic dataset represents a useful resource for the field and reveals important developmental differences between native and cultured neutrophils. A striking difference is the elevated abundance of mitochondrial proteins in cultured neutrophils, which is reflected in active mitochondrial respiration. Interestingly, mitochondria have recently been implicated in promoting neutrophil killing of *S. aureus* [60, 61], potentially explaining why cultured neutrophils maintain the ability to suppress *S. aureus*, despite lacking many granule proteins. In contrast to MPO, we found many other granule proteins to be significantly reduced in the proteome of cultured neutrophils. This paucity of cytoplasmic granule proteins was observed for primary (NE), secondary (lactoferrin) and tertiary (MMP9 and CRISP3) granules, and may explain the defect in *C. albicans* killing. Degranulation has been the least studied antimicrobial response in cultured neutrophils, with only Dick et al*.* reporting reduced primary granule exocytosis in CD34^+^ ex vivo differentiated versus native neutrophils [42], in line with our finding of reduced NE release. It is likely that both findings are explained by perturbations in granule synthesis rather than reduced function of exocytosis machinery. The decrease in granule protein abundance and the maintenance of mitochondrial metabolism are both indicative of incomplete maturation of cultured neutrophils. This is reminiscent of in vitro erythroid culture from CD34^+^ HSPCs, where the majority of differentiated cells produced are immature erythrocytes (reticulocytes), which subsequently mature in circulation [62, 63]. This also means that care is needed when exploring patient phenotypes with such culture models as they may not reproduce all aspects of a disease, in instances where disease consequences manifest in terminally differentiated mature neutrophils.

Ex vivo culture presents opportunities for genome editing that are not possible in neutrophils isolated from peripheral blood. Similar to previous knockout [45, 54] and overexpression efforts [41], we show that CRISPR/Cas9 can be used to modulate gene expression in cultured neutrophils. Nucleofection of ribonucleoprotein negates the need for using plasmid constructs, which we have found can impact neutrophil differentiation. This represents an important advance in the manipulation of cultured neutrophils that will facilitate moving away from use of imperfect immortalised neutrophil-like cell lines and mouse models, which do not fully recapitulate human neutrophil phenotypes and functions [64]. This technique also provides a model system for investigating the molecular bases of neutropenia and neutrophil immunodeficiencies caused by germline mutations.

## Conclusion

Neutrophils produced using our described culture method are reminiscent of immature, bone marrow-mobilised neutrophils. Use of ex vivo differentiation from donor or patient CD34^+^ cells, coupled with genome editing, are useful new tools for increasing our understanding of neutrophil development.

## Materials and methods

### CD34^+^ stem cell isolation

Peripheral blood mononuclear cells (PBMCs) were isolated from apheresis waste products (NHSBT, Filton, Bristol, UK) with ethical approval from NHS Research Ethics committee (REC 18/EE/0265). PBMCs were isolated by density centrifugation using Histopaque 1077 (Sigma Aldrich) as previously described [65, 66]. 8–10 mL of blood from apheresis were mixed with 60 µL citrate-dextrose solution (ACD, Sigma Aldrich). 10 mL of Hanks’ balanced salt solution (HBSS, Lonza) was added to samples and the mixture was layered over 25 mL of room temperature (RT) Histopaque 1077 (Sigma Aldrich) then centrifuged at 400×*g* at RT for 35 min with no brake. The interface layer that contains PBMCs was collected and washed five times with HBSS supplemented with ACD (0.6% v/v, Sigma Aldrich). Finally, cells were resuspended in 10 mL red cell lysis buffer (55 mM NH4Cl, 0.137 mM EDTA, 1 mM KHCO_3_, pH 7.5 in water) and washed once with HBSS supplemented with ACD (0.6% v/v, Sigma Aldrich). CD34^+^ cells were isolated from PBMCs using a Human CD34 MicroBead Kit (Miltenyi Biotec) and LS columns (Miltenyi Biotec) as outlined in the manufacturer’s instructions.

### Neutrophil differentiation

CD34^+^ cells were cultured in IMDM (Biochrom, Source Biosciences, Cambridge UK) supplemented with 10% (v/v) fetal calf serum (FCS, Life Technologies) and 1% (v/v) penicillin/streptomycin (P/S, Sigma Aldrich), at 37 °C in 5% CO_2_. Stem Cell Factor (SCF, 50 ng/mL, Miltenyi Biotech), Flt-3 Ligand (50 ng/mL, Miltenyi Biotech), Interleukin-3 (IL-3, 10 ng/mL, R&D Systems), GM-CSF (10 ng/mL, Miltenyi Biotech) G-CSF (10 ng/mL, Miltenyi Biotech) were introduced at the following times post CD34^+^ isolation: IL-3, SCF and Flt3-L from day 0–3; GM-CSF, IL-3, SCF and Flt3-L from day 3–7; GM-CSF and G-CSF from day 7–10 and G-CSF only from day 10–17. CD34^+^ cells were initially plated at 0.1–0.2 × 10^6^ cells/mL on Day 0. A full media change was completed at Day 3 post CD34^+^ isolation, after which point cultures were supplemented with additional media every 2–3 days to maintain a cell density of 0.5 × 10^6^ cells/mL. Unless stated otherwise all functional assays were completed between Day 17 and Day 19 of culture.

### Native neutrophil isolation from peripheral blood

Blood samples from consented healthy donors at the University of Bristol were collected with ethical approval from NHS Research Ethics committee (REC 18/EE/0265), into EDTA tubes. Neutrophils were isolated using the EasySep™ direct human neutrophil isolation kit (STEMCELL Technologies) as per the manufacturer’s instructions.

### Multi-fluorophore flow cytometry analysis

Flow cytometry analysis was conducted using 5 × 10^5^ Day 17–19 cultured or GCSF-D neutrophils as previously described [21]. Briefly, samples were washed with PBS, resuspended in 0.1% Zombie Aqua live/dead stain in PBS (BioLegend) and incubated for 10 min in the dark at RT. Samples were then incubated in FC Block (BioLegend) diluted in flow buffer (5 mM ETDA and 0.5% bovine serum albumin (BSA) in PBS) on ice for 5 min, after which a master mix of primary antibodies was added and incubated for 30 min on ice protected from light (at the concentrations indicated in Supplemetary Table S2). Samples were washed twice with flow buffer and fixed using 2–4% paraformaldehyde for 20 min at RT. Cells were analysed using a BD X20 Fortessa flow cytometer within 7 days of sample preparation. Appropriate single fluorescence colour compensation controls were conducted in parallel using Invitrogen OneComp eBeads (Thermo Fisher Scientific). At least 10,000 events were recorded per sample, gated on a singlet, live population and data were processed in FlowJo software (version 9).

### *S. aureus* killing assay

2.5 × 10^6^ native neutrophils or Day 18 cultured neutrophils were resuspended in 500 µL HBSS (Lonza) and combined with 5 × 10^5^ *S. aureus* JE2 strain bacteria in HBSS supplemented with 2 mM CaCL_2_, 2 mM MgCL_2_ and 10% pooled human serum (Seqens, 21000P). Samples were incubated at 37 °C with atmospheric CO_2_ levels on a rotator. 50 µL of each sample was taken immediately and thereafter every hour for 4 h and streaked on agar plates. Agar plates were then incubated at 37 °C overnight and then scored for colonies the following day. Killing efficiency was calculated as (number of colonies with neutrophils/number of colonies with serum only).

### *C. albicans* killing assay

C. *albicans* CaSS1 strain was grown overnight in Yeast Extract-Peptone-Dextrose (YPD) medium at 30 °C, 200 RPM in 5% CO_2_ for 16 h. The next day, C. *albican*s concentration was determined by optical density, and yeast were sub-cultured in YPD with 0.05 µg/mL doxycycline for 3 h at 30 °C, 200 RPM in 5% CO_2_. Yeast were resuspended in RPMI-1640 and opsonised with 5% pooled human serum for 30 min before incubating with native or cultured neutrophils at a MOI of 2.5 or 5 at 37 °C for 2.5 h. Samples were treated with 0.1% Triton X-100 (Sigma Aldrich) to lyse neutrophils, washed three times with PBS, incubated with alamarBlueTM (Thermo Fisher Scientific) for 17 h and fluorescence was measured using a FLUOstar Omega Microplate Reader (BMG Labtech) to quantify metabolically active *C. albicans*.

### C. albicans NET assay

C. *albicans* CaSS1 strain was grown overnight in YPD medium at 30 °C, 200 RPM in 5% CO_2_ for 16 h. The next day, C. *albican*s concentration was determined by optical density, sub-cultured in YPD for 3 h at 30 °C, 200 RPM in 5% CO_2_. Fungi were resuspended in RPMI-1640 and opsonised with 10% pooled human serum for 30 min. Fungi were pelleted and plated at 0.5 × 10^6^ in a 24 well plate in 1 mL in NETs media (RPMI1640 supplemented with 0.025% HSA and 10  mM HEPES). Fungi were incubated at 37  C until pseudo hyphae formed. 1 × 10^5^ native or cultured neutrophils were added per well and incubated for 4 h. NETs were visualised as previously described [67]: cells were stained with 1 µM of SYTO green to label all neutrophils and 1 µM SYTOX orange for NETs (Thermo Fisher Scientific) and imaged using an EVOS^®^ FL Cell Imaging System (Thermo Fisher Scientific). NETs were quantified on ImageJ Fiji software.

### Cytokine release

1 × 10^5^ neutrophils were plated in 200 µl in triplicate in RPMI-1640 with phenol red (GIBCO), 10% fetal bovine serum (FBS, Sigma) and 1% P/S (Biochrom) and stimulated with 100 ng/mL bacterial lipopolysaccharide (LPS from *E. coli* O127:B8, Sigma Aldrich) or 5 µM resiquimod (Sigma Aldrich) overnight at 37  C in 5% CO_2_. IL-8 and IL-6 levels in the resulting supernatants were measured using Human IL8/CXCL8 DuoSet ELISA and Human IL-6 DuoSet kits following the manufacturers protocol (both R&D Systems).

### Zymosan degranulation assay

1 × 10^5^ neutrophils were plated in duplicate, in 200 µL RPMI-1640 (GIBCO) supplemented with 0.025% human serum albumin (HSA; Sigma-Aldrich) and 10 mM HEPES (Sigma-Aldrich) and were stimulated with 25 µg/mL opsonised Zymosan (Sigma Aldrich). After 1 hour, samples were centrifuged at 300×*g*, 100 µL of supernatant was removed from each well and the NE concentration measured using a human Neutrophil Elastase/ELA2 DuoSet ELISA kit (R&D Systems) as per the manufacturer’s protocol.

### Reactive oxygen species production

1 × 10^5^ neutrophils were plated in 100 µL of ROS media (HBSS with 10 mM HEPES and 0.025% HSA, both Sigma Aldrich) in a white, 96 well plate and incubated at 37 °C, 5% CO_2_ for 15 min at which point horseradish peroxidase (HRP, 200 U/ml, Sigma Aldrich) and luminol (25 µM, Sigma Aldrich) were added at 1:200. Following a 15-min incubation at 37 °C, neutrophils were stimulated with a final concentration of the indicated stimulant and chemiluminescence was recorded for 3 h in 2.5-min intervals using a FLUOstar plate reader (BMG Labtech).

For APF experiments, 1 × 10^5^ neutrophils were plated in 100ul of ROS media supplemented with 10 µM APF (Thermo Fisher) in black, clear, flat-bottomed plates and cells were incubated at 37 °C, 5% CO_2_ for 45 min. After incubation, cells were centrifuged at 400×*g* for 5 min and media was aspirated and replaced with plain ROS media. Neutrophils were stimulated with 100 nM PMA (Sigma Aldrich), and fluorescence measured every 2.5 min for 4 h using a BMG FLUOstar plate reader (emission 490 nm, excitation 515 nm).

### TMT mass spectrometry

Day 18 cultured neutrophils and native neutrophils were isolated or cultured from 3 separate donors as described above. Sample pairs were donor-matched, with CD34^+^ cells and native neutrophils isolated from the same fresh apheresis cone. Neutrophils were sorted using CD66b expression using a BD Influx Cell Sorter (BD Biosciences). Cells were immediately pelleted and lysed in supplemented RIPA buffer (EDTA 10 mM, 50 mmol/l TCEP (Sigma Aldrich), 2 mM PMSF protease inhibitor, 1/50 v/v Protease Inhibitor Cocktail Set V (Calbiochem) and flash-frozen in liquid nitrogen for storage until later analysis. All samples were then thawed on ice, sonicated to fragment DNA, and measured for protein concentration with a Pierce^®^ BCA Protein Assay Kit (Thermo Scientific, cat no 23227) according to the manufacturer’s instructions. Due to TCEP being used during cell lysis, a reducing agent-compatible kit was used. 100 µg of each sample were then digested with trypsin and labelled with TMT reagents according to the manufacturer’s protocol (Thermo Fisher Scientific). The resulting peptides were identified by nano LCMS/MS with a Orbitrap Fusion Tribrid Mass Spectrometer (Thermo Fisher Scientific). Raw files were analysed using Proteome Discoverer software v. 2 and cross-referenced against the human UniProt database (human). PD analysis was conducted for full trypsin digestion, removing all hits with more than one missed cleavage. All peptides were filtered to meet an FDR of 1%. Log_2_ fold changes (Log2FC) were calculated between cultured and native neutrophils to identify differentially expressed proteins (|Log_2_FC|> 1, P value < 0.05). Log_2_FC volcano plots were generated using Microsoft Excel. Log_2_FC clustering analysis and the resulting heatmap visualisations were generated using R v. 4.2.2. Protein–protein interaction networks for differentially expressed proteins were generated using the STRING database and Cytoscape v. 3.9.1.

### CRISPR/Cas9 gene editing

Nucleofection of ribonucleoproteins (RNP) was completed using the Nucleofector 4D (Lonza) using a P3 Primary Cell 4D-NucleofectorTM X Kit S (Lonza) and Invitrogen™ TrueCut™ Cas9 Protein v2 (Thermo Fisher Scientific) following the manufacturer’s recommended protocols. CD34^+^ cells were isolated and cultured as described above until Day 3 of culture. 0.3 × 10^6^ cells were transduced per reaction. 50 pmol of Cas9 was mixed with 125 pmol of gRNA (62.5 pmol of two guides with the same gene target or a scrambled (SCR) gRNA control) per reaction and incubated at 25 °C for 15 min to form RNPs, which were stored on ice for up to 4 h. All guides were designed using the Synthego CRISPR Design Tool and produced by Synthego (Redwood City, USA). Supplemented Nucleofector Solution (SNS) was made up fresh at a 4.5:1 ratio of nucleofector solution to supplement. 0.3 × 10^6^ Day 3 cells were spun down, washed in PBS, and resuspended in 20 µL SNS. Cells were added to RNPs, mixed gently, and transferred to a nucleocuvette cassette. Cells were then electroporated using the manufacturers recommended Nucleofector 4D program EO-100. 80 µL of prewarmed 37 °C StemSpan was dripped gently into each reaction to dilute the SNS. Cells were replated in 2 mL of StemSpan (Stem Cell Technologies) supplemented with 1% (v/v) P/S (Sigma Aldrich), SCF (50 ng/mL, Miltenyi Biotech), Flt3-L (50 ng/mL, Miltenyi Biotech), IL-3 (10 ng/mL, R&D Systems), GM-CSF (10 ng/mL, Miltenyi Biotech) and GCSF (10 ng/mL, Miltenyi Biotech). Cells were allowed to recover for 48 h, after which they were cultured from Day 5 as indicated in the neutrophil culture methodology section (Fig. 1A).

### Seahorse metabolic flux analysis

Seahorse XFe 96 calibration plates were hydrated in 200ul culture grade water in a non-CO_2_ incubator overnight. Water was replaced with pre-warmed XF calibrant and incubated in a non-CO_2_ incubator for at least 45 min prior to calibration. 4 × 10^5^ cells were plated in 180 µL Seahorse media (Seahorse XF DMEM medium, 5 mM glucose, 2 mM glutamine). 20 µL of 10X oligomycin (final concentration 1.5 µM) was added to port A, 22.2 µL of FCCP (final concentration 500 nM) in port B and 24.7 µL of AA/RO into port C (final concentration 1 µM). The injection plate was overlayed on the calibration plate and inserted into the Seahorse XFe 96 analyser for calibration. The culture plate with seeded neutrophils was later exchanged after calibration. 3 basal reads were acquired before the first injection and reads were obtained every 6 min thereafter.

### Statistical analysis

Data was organised and analysed using GraphPad Prism 8 software. Mean ± SD is plotted for a minimum of n = 3 differentiations unless otherwise stated. Statistical analysis was completed where appropriate using Student’s t test when comparing two different samples, or one-way ANOVA where multiple samples were being examined, with asterisks on the graph represent the following: *P < 0.05, **P < 0.01, and ***P < 0.001.

## Supplementary Information

Supplementary material 1.

Supplementary material 2

## Data Availability

The mass spectrometry data have been deposited in the ProteomeXchange Consortium via the PRIDE partner repository with the dataset identifier PXD052008. The research materials supporting this publication can be accessed by contacting corresponding authors.
