# Correction to: Neutrophils cultured ex vivo from CD34 + stem cells are immature and genetically tractable

**DOI:** 10.1186/s12967-024-05644-3

**Published:** 2024-10-04

**Authors:** Claire A. Naveh, Kiran Roberts, Przemysław Zakrzewski, Christopher M. Rice, Fernando M. Ponce-Garcia, Kathryn Fleming, Megan Thompson, Nawamin Panyapiean, Huan Jiang, Stephanie Diezmann, Pedro L. Moura, Ashley M. Toye, Borko Amulic

**Affiliations:** 1 School of Cellular and Molecular Medicine, Biomedical Sciences Building, University of Bristol, Bristol, BS8 1TD UK; 2 School of Biochemistry, Biomedical Sciences Building, University of Bristol, Bristol, BS8 1TD UK; 3 Center for Hematology and Regenerative Medicine, Department of Medicine Huddinge (MedH), Karolinska Institutet, Huddinge, Sweden

**Naveh et al. Journal of Translational Medicine (2024) 22:526**

10.1186/s12967-024-05337-x

Following publication of the original article [1], we have been notified that the supplementary information link leads only to the figures’ legends, but not to figures.

The original article was updated.
