# Supplementary material for: Neutrophils cultured ex vivo from CD34+ stem cells are immature and genetically tractable
Source: J Transl Med. 2024 May 31;22:526. doi: 10.1186/s12967-024-05337-x (PMC11143668; doi:10.1186/s12967-024-05337-x)
Supplement: Supplementary file 1 — Supplementary material 1. [file 12967_2024_5337_MOESM1_ESM.pdf]

Supplemental Figures:

Fig. S1 Additional characterisation of cultured neutrophils.

A: Schematic outlining culture procedure (top) or acquisition of native neutrophils. Figure generated in Biorender. B: Representative gating strategy of native and cultured cells. Native cells were gated on a singlet, appropriately sized population where monocytes and eosinophils were gated out using CD14 and IL-5R expression respectively. Cultured neutrophils were gated on a singlet, appropriately sized, live population using granulocyte markers CD66b and CD15. C: Quantitative description of nucleation in cultured neutrophils at day 17 of differentiation, n=3. D: Percentage of culture classified as $CD14^{high}$ (monocytes) and $CD14^{low}$ (neutrophils) by flow cytometry at day 17 of differentiation, n=3. E: CD14 MFI measured by flow cytometry at day 17 of culture, n=3. F: Analysis of viability by flow cytometry at days 17-21 of differentiation, live= annexin V-; PI -; dead: annexin V+; PI+, n=1. G: Percent of cells expressing CD66B, measured by flow cytometry, at late stage of the culture protocol, n=2-3.

Fig. S1

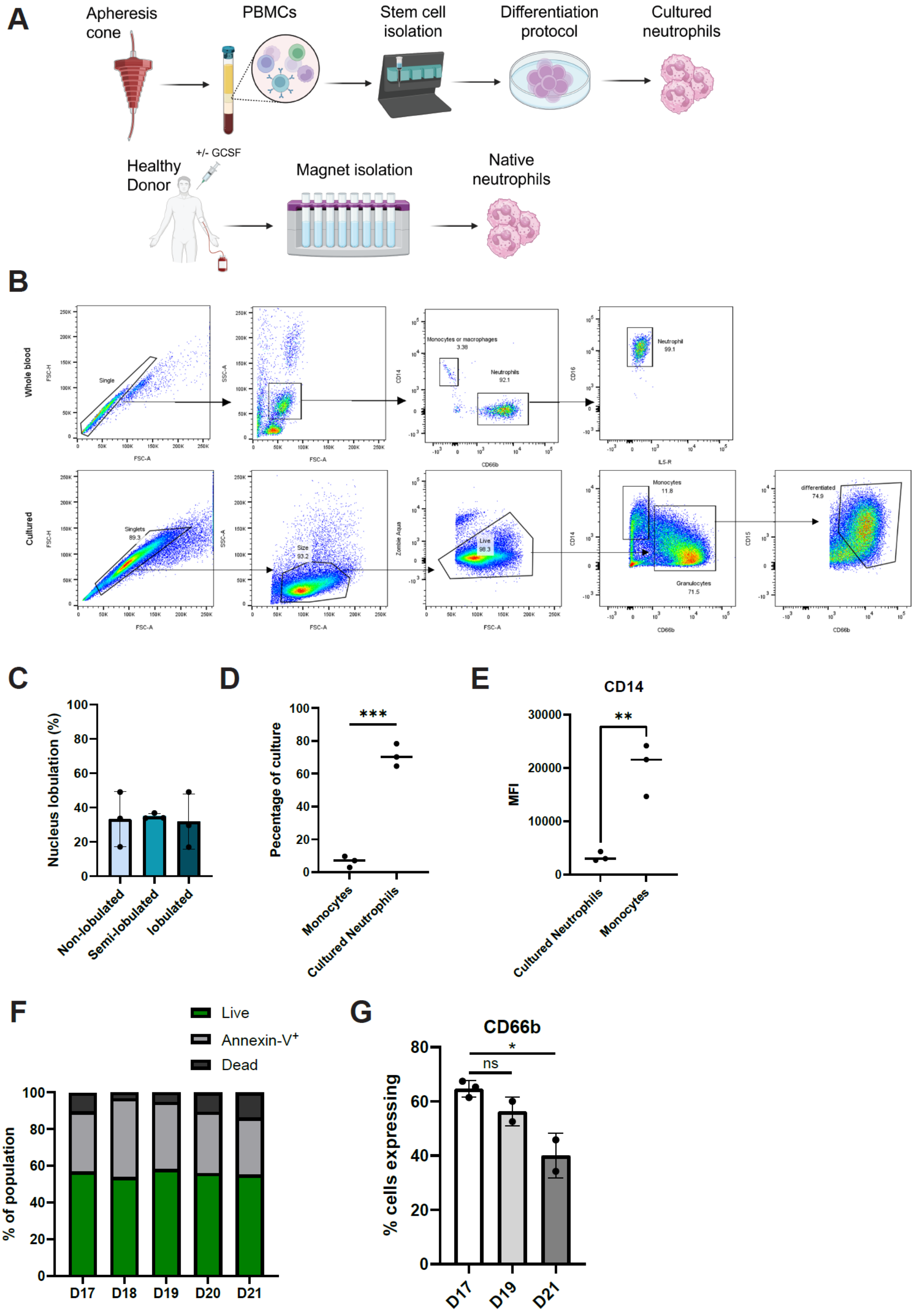
A
Apheresis cone
PBMCs
Stem cell isolation
Differentiation protocol
Cultured neutrophils
Healthy Donor
+/- GCSF
Magnet isolation
Native neutrophils
B
Whole blood
Cultured
Single
Monocytes or macrophages
3.38
Neutrophils
92.1
Neutrophil
99.1
Singlets
89.3
Size
93.2
Live
98.3
Monocytes
11.8
Granulocytes
71.5
differentiated
74.9
FSC-A
FSC-H
SSC-A
CD14
CD66b
CD16
IL5-R
Zombie Aqua
CD15
C
Nucleus lobulation (%)
Non-lobulated
Semi-lobulated
lobulated
D
Pecentage of culture
***
Monocytes
Cultured Neutrophils
E
CD14
MFI
**
Cultured Neutrophils
Monocytes
F
Live
Annexin-V+
Dead
% of population
D17
D18
D19
D20
D21
G
CD66b
% cells expressing
ns
*
D17
D19
D21

Fig. S2 Native and GCSF-D neutrophil staining properties and purity

A: Representative scatter plots of neutrophils gated on size, CD45, CD15 and CD66b expression. B-C: CD101 (B) and CD10 (C) expression in peripheral blood neutrophils from native (red), GCSF-D (blue) and cultured neutrophils (black), with fluorescence minus one (FMO) controls for cultured neutrophils and peripheral blood neutrophils displayed in grey. D: Percentage of cells expressing CD10, in native, GCSF-D and cultured (day 17) neutrophils, n=3-4. E-G: Representative FACS plots of CD15 (F), CD66B (G) and CD11b (H), including unstained controls. H: Representative images of NETs induced by 10 uM A23187, stained with SYOTO Green and SYTOX Orange. I: Quantification of I, n=2-3.

Fig. S2

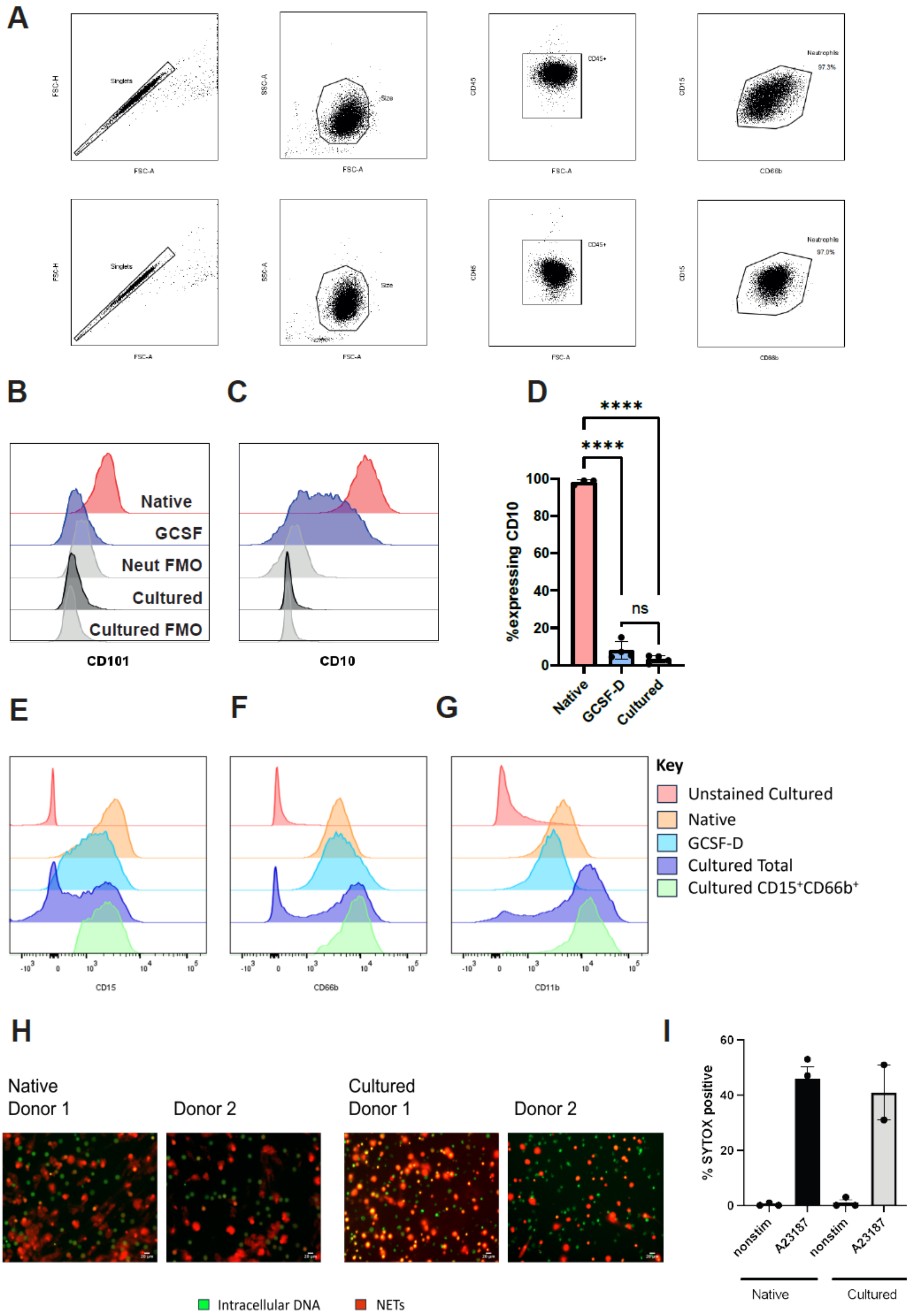
A
Singlets
FSC-H
FSC-A
SSC-A
Size
CD45
CD45+
CD15
CD66b
Neutrophils
B
Native
GCSF
Neut FMO
Cultured
Cultured FMO
CD101
C
CD10
D
****
****
ns
%expressing CD10
100
80
60
40
20
0
Native
GCSF-D
Cultured
E
F
G
CD15
CD66b
CD11b
Key
Unstained Cultured
Native
GCSF-D
Cultured Total
Cultured CD15⁺CD66b⁺
H
Native
Donor 1
Donor 2
Cultured
Donor 1
Donor 2
Intracellular DNA
NETs
I
% SYTOX positive
60
40
20
0
nonstim
A23187
nonstim
A23187
Native
Cultured

Fig. S3 Cultured neutrophils differ in mitochondrial and granule protein abundance

A: Representative sorting gates for native and cultured neutrophils before TMT mass spectrometry. B: Top 8 unique Reactome 2022 pathway terms of enriched (red) and under-represented (blue) genes using the GO Biological Pathway module. C: Normalised protein abundances of key TCA cycle and respiratory electron transport proteins, n=3 differentiations. D: Normalised abundances of key granule proteins quantified between cultured and primary neutrophils in the dataset reported in this work (n=3, Naveh C *et al.*) and between GCSF-treated neutrophils and neutrophils from healthy donors (n = 3, data from Aymonnier K *et al*. 2024). An inter-dataset comparison (comparing Log_2FC of both datasets) is highlighted above the data; intra-dataset comparisons against baseline are highlighted below the data. ns = no significant difference; * = $P < 0.05$; ** = $P < 0.01$. E-F: Enriched (E) and under-represented (F) proteins processed using the STRING database using a medium confidence setting to produce protein-protein interaction networks. Clusters are shown in different colours: E) ribosomal proteins in red, mitochondrial proteins in blue and granule proteins in green and in F) innate immune system and degranulation in red, mRNA processing in blue and chromatin organisation in green.

Fig. S3

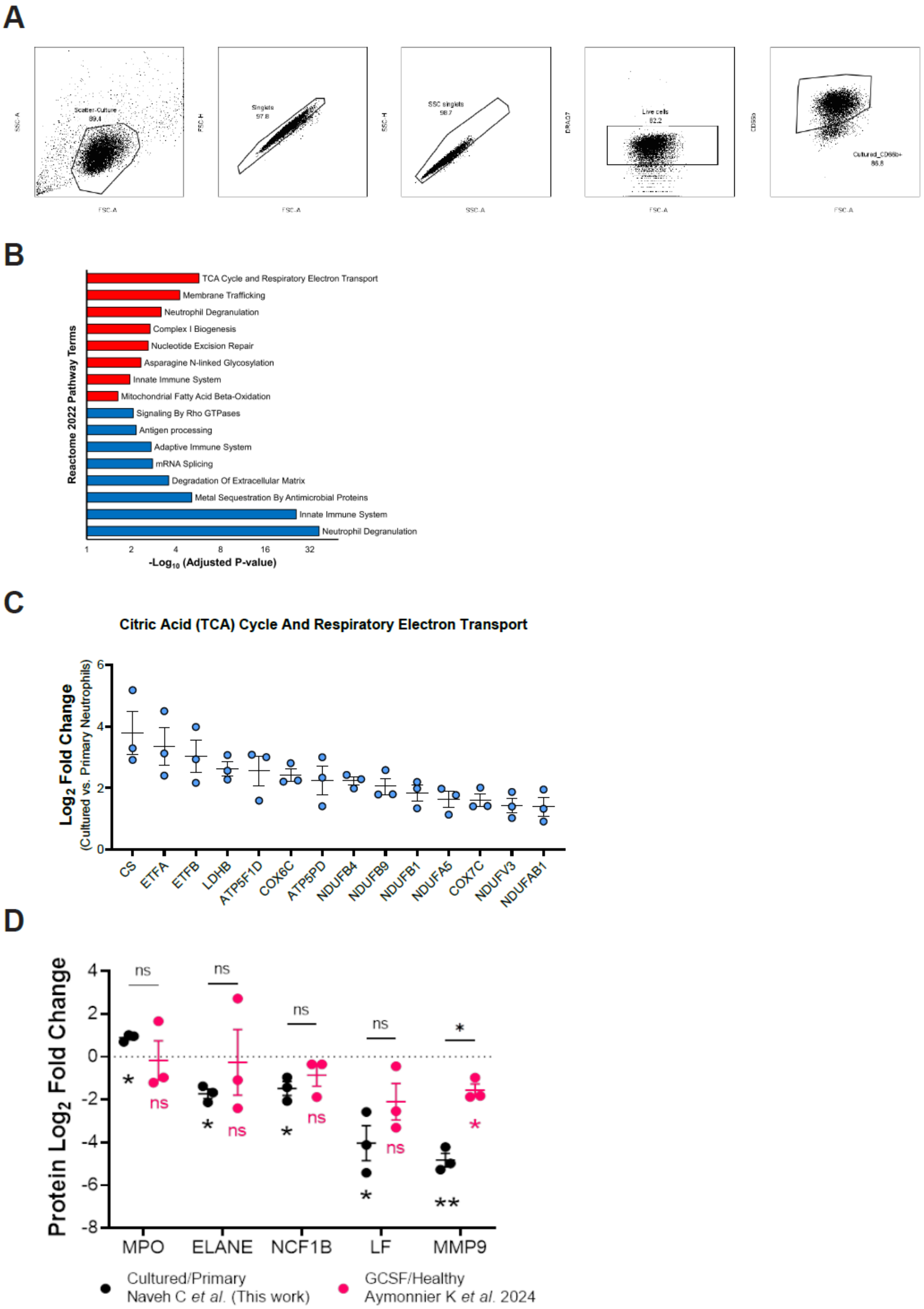
A
B
Reactome 2022 Pathway Terms
TCA Cycle and Respiratory Electron Transport
Membrane Trafficking
Neutrophil Degranulation
Complex I Biogenesis
Nucleotide Excision Repair
Asparagine N-linked Glycosylation
Innate Immune System
Mitochondrial Fatty Acid Beta-Oxidation
Signaling By Rho GTPases
Antigen processing
Adaptive Immune System
mRNA Splicing
Degradation Of Extracellular Matrix
Metal Sequestration By Antimicrobial Proteins
Innate Immune System
Neutrophil Degranulation
1
2
4
8
16
32
-Log10 (Adjusted P-value)
C
Citric Acid (TCA) Cycle And Respiratory Electron Transport
Log2 Fold Change
(Cultured vs. Primary Neutrophils)
6
4
2
0
CS
ETFA
ETFB
LDHB
ATP5F1D
COX6C
ATP5PD
NDUFB4
NDUFB9
NDUFB1
NDUFA5
COX7C
NDUFV3
NDUFAB1
D
Protein Log2 Fold Change
4
2
0
-2
-4
-6
-8
ns
ns
ns
ns
*
*
ns
*
ns
*
ns
*
ns
*
**
*
MPO
ELANE
NCF1B
LF
MMP9
Cultured/Primary
Naveh C et al. (This work)
GCSF/Healthy
Aymonnier K et al. 2024

E

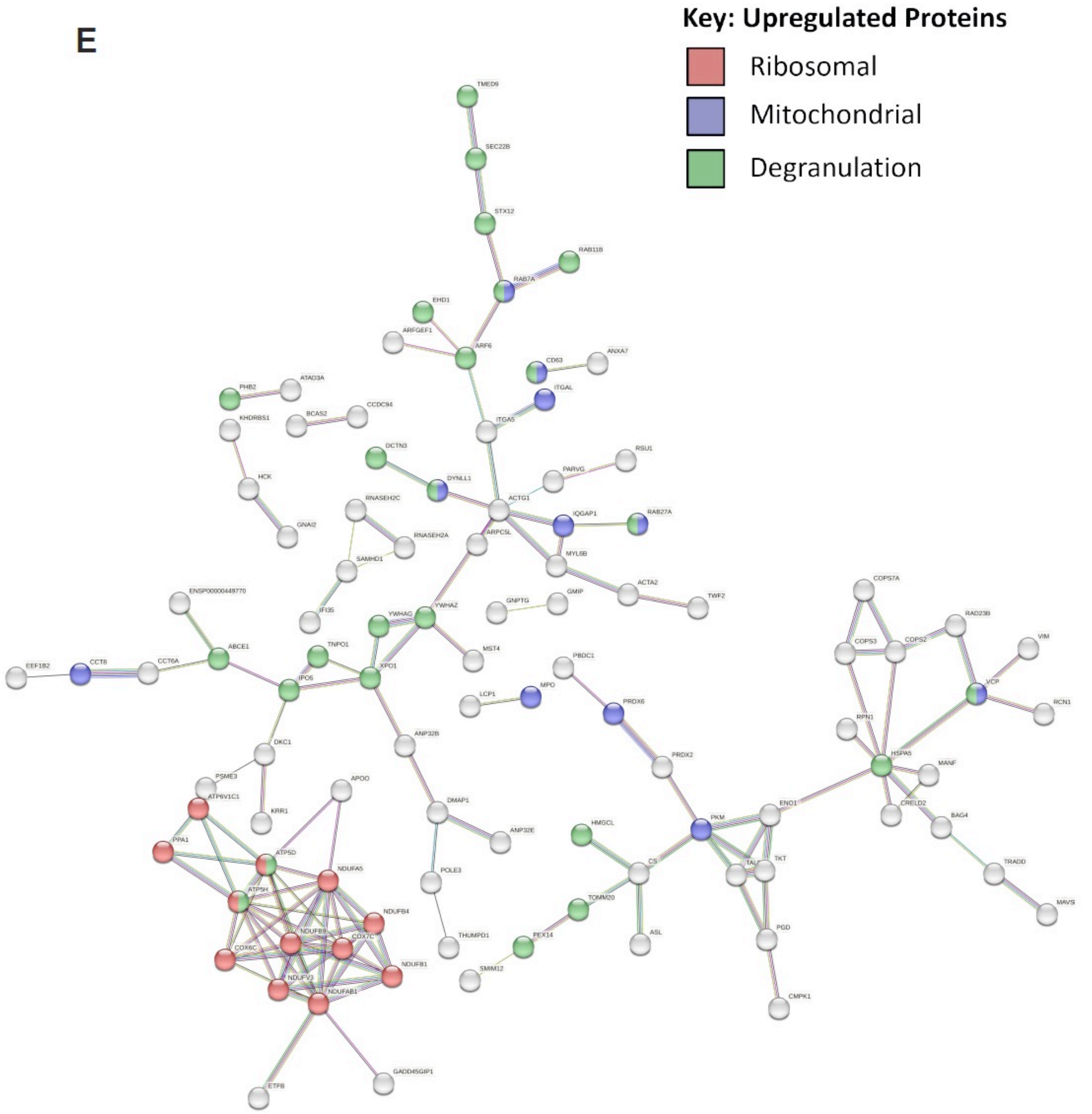
Key: Upregulated Proteins
Ribosomal
Mitochondrial
Degranulation
TMED9
SEC22B
STX12
RAB11B
RAB7A
EHD1
ARFGEF1
ARF6
CD63
ANXA7
ITGAL
ITGA5
PHB2
ATAD3A
KHDRBS1
BCAS2
CCDC94
DCTN3
DYNLL1
ACTG1
PARVG
RSU1
IQGAP1
RAB27A
MYL6B
ACTA2
TWF2
ARPC5L
HCK
GNAI2
RNASEH2C
RNASEH2A
SAMHD1
IFI35
ENSP00000449770
ABCE1
CCT6A
CCT8
EEF1B2
TNPO1
IPO5
XPO1
YWHAG
YWHAZ
MST4
GNPTG
GMIP
PBDC1
PRDX6
PRDX2
LCP1
MPO
DKC1
PSME3
KRR1
ANP32B
DMAP1
ANP32E
POLE3
THUMPD1
APOO
ATP6V1C1
PPA1
ATP5D
ATP5H
NDUFA5
NDUFB4
NDUFB9
COX7C
COX6C
NDUFB1
NDUFV3
NDUFAB1
ETFB
GADD45GIP1
HMGCL
CS
TOMM20
PEX14
SMIM12
ASL
PKM
ENO1
TKT
PGD
CMPK1
COPS7A
COPS3
COPS2
RAD23B
VIM
VCP
RCN1
RPN1
HSPA5
MANF
CRELD2
BAG4
TRADD
MAVS

F

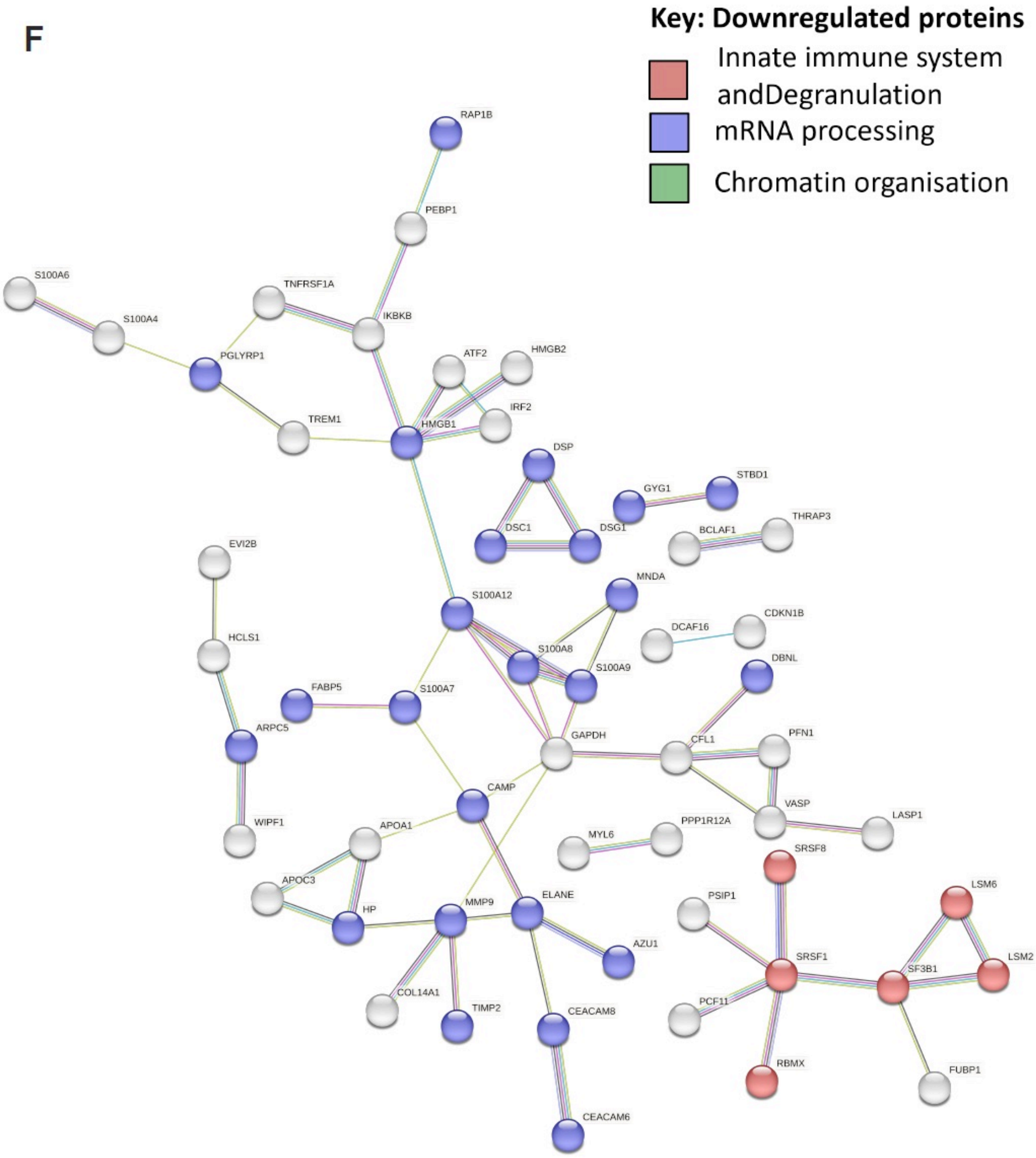
Key: Downregulated proteins
Innate immune system andDegranulation
mRNA processing
Chromatin organisation
RAP1B
PEBP1
S100A6
TNFRSF1A
S100A4
IKBKB
PGLYRP1
ATF2
HMGB2
IRF2
TREM1
HMGB1
DSP
GYG1
STBD1
DSC1
DSG1
BCLAF1
THRAP3
EVI2B
MNDA
S100A12
HCLS1
DCAF16
CDKN1B
S100A8
S100A9
DBNL
FABP5
S100A7
ARPC5
GAPDH
CFL1
PFN1
CAMP
VASP
LASP1
WIPF1
APOA1
MYL6
PPP1R12A
SRSF8
APOC3
HP
MMP9
ELANE
PSIP1
LSM6
AZU1
SRSF1
SF3B1
LSM2
COL14A1
TIMP2
CEACAM8
PCF11
RBMX
FUBP1
CEACAM6

Fig. S4 CD66b expression and viability is unchanged by CRISPR/Cas9 mediated knockout in cultured neutrophils

A: Percentage of PI positive cells in Scr vs β2M (left) and CD11b (right) KO cells respectively by flow cytometric surface marker staining on day 7 of culture. B: Percentage of CD66b expressing cells in β2M (left) and CD11b (right) CRISPR/Cas9 KO cells, by flow cytometric surface marker staining on day 17 of culture.

Fig. S4

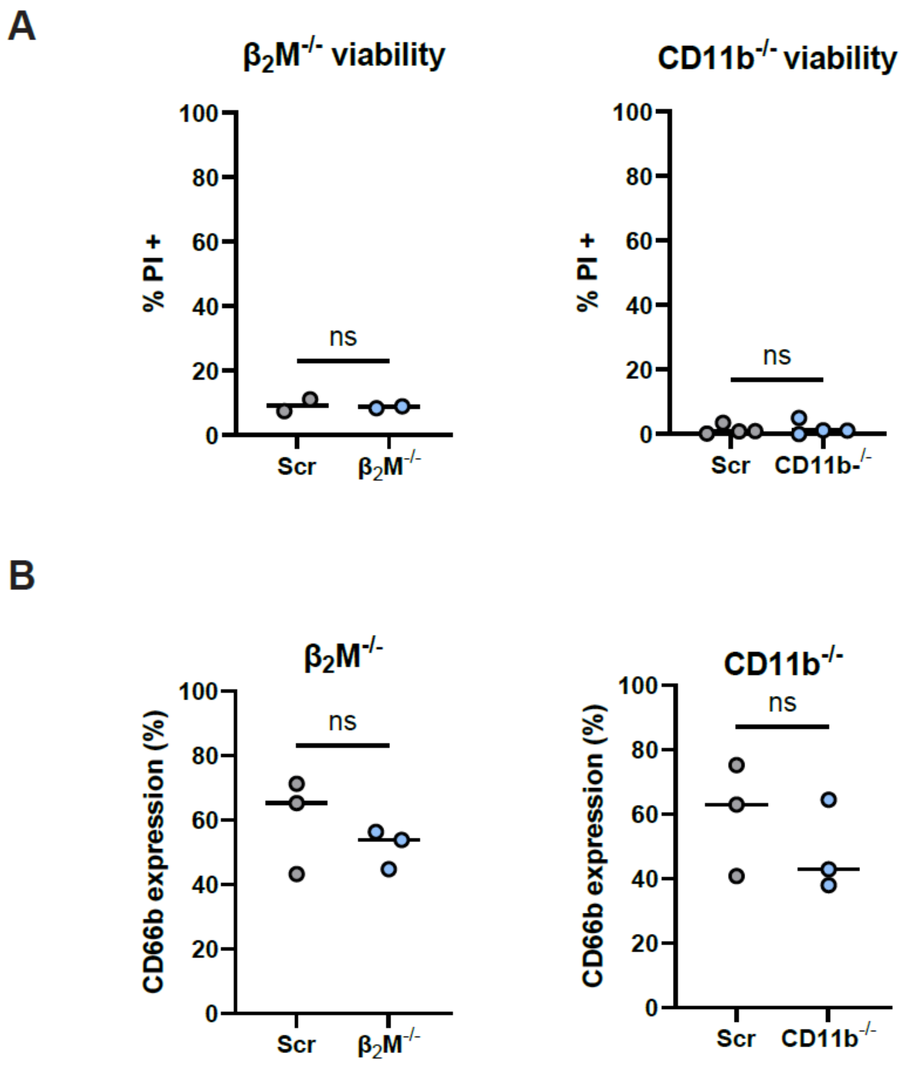

Supplemental Methods

Cytospin preparation and imaging

1x10^5 cells were removed from cultures at the indicated timepoints post CD34$^+$ isolation. Cells were washed with PBS and spun onto glass slides at 1000xg for 5 minutes (Thermo Scientific Cytospin). Samples were then fixed in 100% methanol for 10 minutes and then stained with May Gruwald–Giesma stains as per the manufacturer's instructions (Merck).

Four fluorophore flow cytometry analysis

1x10^5 cells were removed on day 8 of cell culture and labelled with conjugated antibodies for 25 minutes at 4˚C, using commercial nonspecific IgG controls for nonspecific staining. Propidium iodide labelling was used to identify the dead cell population. Samples were analysed using a MacsQuant flow cytometer (Miltenyi Biotec) and processed using FlowJo software (Version 9).

Viability assay

Positive selection of CD66b+ cells was performed using a CD66abce MicroBead Kit (Miltenyi Biotech) following the manufacturer's instructions. After the selection, CD66b-expressing cells were returned to culture in IMDM with the addition of G-CSF.

2.0x10^5 cells were washed with PBS and resuspended in 100µL Annexin V binding buffer (BioLegend) and stained with FITC-Annexin V (BioLegend) for 15 min at RT. DRAQ7™ (BioLegend) was added directly before the cells were analyzed using NovoCyte 3000.

Supplemental Tables:

Supplementary Table 1: Proteins uniquely expressed in cultured neutrophils and not native neutrophils.

**Gene name**	**Gene description**
ANXA6	Annexin A6
DNAJC7	DnaJ homolog subfamily C member 7
EIF5	Eukaryotic translation initiation factor 5
DKFZp686E1893	Putative uncharacterized protein DKFZp686E1893
CCNB2	G2/mitotic-specific cyclin-B2
SELENOT	Selenoprotein T
ARL2	ADP-ribosylation factor-like protein 2
SPCS3	Signal peptidase complex subunit 3
PPDPF	Pancreatic progenitor cell differentiation and proliferation factor
MT1G	Metallothionein-1G
PNKD	Probable hydrolase PNKD
KCNK1	Potassium channel subfamily K member 1

Supplementary Table 2: Antibodies and fluorescent dyes used for flow cytometry

Receptor	Fluorophore conjugated/ Channel	Final concentration	Company	Catalogue Number
CD16	FITC	1/50	Biolegend	302006
CD15	Alexa700	1/50	Biolegend	301920
CD66b	APC	1/50	Biolegend	305118
CD66b	APC-Cy7	1/50	Biolegend	305126
CD11b	PE-Cy7	1/50	Biolegend	301412
CD10	PE	1/50	Biolegend	312204
CD62L	PECy7	1/50	Biolegend	304822
CXCR2	PE-DAZZLE	1/50	Biolegend	320722
CD101	PerCP Cy5.5	1/50	Biolegend	331016
CD14	Brilliant Violet 785	1/50	Biolegend	367122
CD125 (IL5-Ra)	Biotin	1/25	Miltenyi Biotec	130-110-543
CD34	VioBlue	1/50	Miltenyi Biotec	130-113-182
B2M	APC	1/50	Biolegend	316311
Zombie Aqua	BV510	1/1000	Biolegend	423101
DRAQ7	-	1/200	Biolegend	424001
Annexin V	FITC	1/20	Biolegend	640906

Supplementary Table 3: Synthego guide RNA for CRISPR-Cas9 gene editing

CRISPR guide sequences		Antibody used to assess efficiency
B2M #1	GGCCGAGAUGUCUCGCUCCG	APC β2-microglobulin (BioLegend)
B2M #2	UCACGUCAUCCAGCAGAGAA	APCβ2-microglobulin (BioLegend)
CD11b #1	UAGCCUUGACCUUAUGUCAU	APC CD11b (BioLegend)
CD11b #2	CAGAGCGUGGUCCAGCUUCA	APC CD11b (BioLegend)
SCR #1	(Sequence not provided)	n/a
SCR #2	(Sequence not provided)	n/a
